# Evaluation of Prescription Adaptation Following Changes to A Provincial Drug Insurance Formulary

**DOI:** 10.3390/pharmacy7010006

**Published:** 2019-01-07

**Authors:** Robert T. Pammett

**Affiliations:** 1 Northern Health Authority, Prince George, BC V2L 5B8, Canada; Robert.Pammett@northernhealth.ca; 2 Faculty of Pharmaceutical Sciences, University of British Columbia, Vancouver, BC V6T 1Z3, Canada; robert.pammett@ubc.ca

**Keywords:** managed care, inter-professional collaboration, primary care

## Abstract

On 1 December 2016, British Columbia’s (BC) provincial drug insurance program changed which medications in certain classes would benefit under the insurance program in an attempt to reduce expenditure. As part of the modernization, HMG-CoA reductase inhibitors (Statins), Angiotensin converting enzyme inhibitors (ACEI), angiotensin receptor blockers (ARB), and dihydropyridine calcium channel blockers (CCB) were affected. Prescribers and pharmacists had six months to discuss the changes with patients, and change medications if deemed necessary. **Purpose:** To quantify the changes made to prescriptions and to adjust to the Modernized Reference Drug Program. **Methods:** A retrospective chart review was conducted at two clinics in Prince George, BC. Charts for patients that were prescribed any drugs in the affected classes were reviewed to determine if, and when, they had been changed, and by which health care professional. In December 2016, a clinical pharmacist, integrated within the study clinics, informed prescribers of the changes, and made patient-specific clinical notes within the charts. The notes described the changes and recommended alternative agents and appropriate dosing in order to assist the prescriber to have a conversation with the patient regarding the switch. **Results:** Out of 429 unique patients, 233 patients were prescribed a Statin, 229 patients an ACEI, 110 an ARB and, 83 a CCB. Sixty-five drug changes were indicated to reflect the modernization, and with guidance from a clinical pharmacist, nurse practitioners (NPs), and family physicians (FPs), 65% of these identified drugs were switched to reflect the modernization. Community pharmacists made no drug changes in the study sample, despite the prescriptive authority and compensation available to do so. Province-wide, approximately 21% to 33% of affected drugs were switched during the same time-frame. Direct collaboration between a clinical pharmacist, working alongside NPs and FPs, was more successful in optimizing these medications when compared to standard practice, or community pharmacists alone.

## 1. Introduction

British Columbia (BC), Canada, has a provincially funded prescription drug insurance program called Pharmacare. Pharmacare includes a number of plans, which covers many prescription medications for eligible beneficiaries. However the most common plan incorporates an income tested deductible which must be paid prior to receiving medication at a reduced cost for beneficiaries. The reference drug program (RDP) has been part of Pharmacare for over two decades [1], and applies to a number of therapeutic drug classes where the evidence supports the drugs within these classes to be equally safe and effective. Through the RDP, Pharmacare fully covers the selected drug(s), which are the most cost effective within these drug classes for its beneficiaries. As of 1 December 2016, the RDP underwent a modernization which changed the medications within these classes that would be eligible for full benefit as part of Pharmacare. Due to an increase in the number of generic drugs available in these classes, as well as generic drug pricing reforms in Canada, these changes are expected to save the provincial insurer $27 M CAD by the year 2020.

As part of the modernization, HMG-CoA reductase inhibitors (Statins), angiotensin converting enzyme inhibitors (ACEIs), angiotensin receptor blockers (ARBs), and dihydropyridine calcium channel blockers (CCBs) were significantly affected. The drugs, which would become the full benefits under the modernized RDP, were atorvastatin and rosuvastatin (Statins), amlodipine (CCB), candesartan, losartan, telmisartan and valsartan (ARBs), and ramipril (ACEI). Providers (pharmacists and prescribers) were informed of the pending changes via posted communication packages. They were given from 1 June 2016 until 30 November 2016 (the “transition period”) to discuss the modernization of the program with affected patients and make any indicated therapeutic substitutions prior to the formulary changes taking effect. It is within the scope of pharmacists working in dispensaries in British Columbia to adapt (the official nomenclature for changing prescriptions for pharmacists in the province) these prescriptions, changing drugs within these classes through a therapeutic substitution to the low cost reference drug independently from other prescribers if deemed appropriate [2]. However pharmacists working outside of the dispensary setting may not independently adapt prescriptions as they do not possess the original prescription, which is retained at a dispensing pharmacy.

The purpose of this study was to determine the extent to which providers (retail pharmacists, FPs, or NPs) would adapt prescriptions to reflect this change in formulary, and the extent to which adaptations would occur during both the six-month transition period and the six months following the transition.

## 2. Methods

A retrospective chart review in two primary care practices, that was composed of prescribing NPs and FPs, and an associated, integrated, non-dispensing, clinical pharmacist (RTP), was conducted in Prince George, British Columbia in December 2016 to determine the extent to which providers adapted prescriptions in response to the modernized RDP during the transition period. Prescription records in charts were reviewed for all patients that were prescribed Statins, ACEIs, ARBs, and dihydropyridine CCBs in order to determine if the patient was prescribed a drug which would be a full benefit under the modernized RDP, or if they were prescribed a molecule which was no longer a full benefit. Charts were also reviewed to determine if there was a compelling reason to continue a non-RDP drug (such as intolerance to the RDP drug), and if the non-RDP drug already had Special Authority in place (a formal, documented process for accepting a non-RDP drug as a full benefit for that patient due to specific circumstances, such as adverse reactions or lack of efficacy to the RDP drug) [3]. To identify adaptations made by community pharmacists, notifications of the adaptation were used as the surrogate for prescription records. Policy from the British Columbia College of Pharmacists (which governs pharmacist practice in the province) require pharmacists who adapt a prescription to notify the original prescriber and the patient’s most responsible clinician of the change (if these clinicians are different people). The policy indicates that the preferred method of notification is via fax, and as such, the charts were reviewed for this fax notification, or an indication that the notification had been made via some other communication method (such as a phone call or posted mail).

At the point of data collection in December 2016, for any non-RDP drug that was identified in the charts, the clinical pharmacist placed an alert within the electronic medical record (EMR) to prompt and guide prescribers within the primary care clinic to an appropriate therapeutic substitution. This included, which therapeutic agent would be a full benefit under the modernized RDP, the approximate equivalent dose, and the recommended regimen, supported by the guidance documents provided by the British Columbia Ministry of Health for this purpose [1]. This action was meant to facilitate the discussion between prescribers and patients when they presented to the clinic.

In September 2018, the same charts were reviewed to determine if medication changes had occurred to reflect the new formulary either during the transition period (1 June 2016 to 1 December 2016), or between 1 December 2016 and 31 May 2017, where health care professional initiated the prescription change.

The data were collected and analyzed using Microsoft Excel (2013) and reported as descriptive statistics.

This research was approved by the University of British Columbia Research Ethics Board, and Northern Health’s Research Review Committee.

## 3. Results

The chart reviews identified a total of 429 unique patients at the two clinics, who were taking at least one drug from the affected classes during the study period. Of these, 233 patients were prescribed a Statin, 229 were prescribed an ACEI, 110 were prescribed an ARB, and 83 were prescribed a dihydropyridine CCB. Prior to the 1 December 2016 formulary change, modernized RDP drugs were the most commonly prescribed drugs within their classes; prescribed 84% (CCB) to 91% (Statins) of the time. There were five instances where the patients had a specific indication for the use of a non-modernized RDP drug and were considered as inappropriate to switch to the modernized RDP drug. A total of 65 instances of prescribed non-modernized RDP drugs were identified, where the patient may have benefited from a switch to the RDP drug.

Six medication agent changes were made during the transition period, and 36 changes were made between 1 December 2016 and 1 June 2017, accounting for 65% of the total potentially indicated medication alterations. All changes were made by FPs or NPs at the primary care clinics; no prescription adaptations, to reflect the change in formulary, were identified as being initiated by community pharmacists from 1 June 2016 to 31 May 2017. See Table 1 for further details.

## 4. Discussion

A limited number of medication changes occurred during the six-month transition period. The majority of what were considered appropriate changes to medications, to reflect the change in formulary, occurred between 1 December 2016 and 31 May 2017. Eighty-eight percent of the changes occurred after the alert within the EMR was set. This alert was meant to support a conversation between prescribers and affected patients regarding the issue. It also provided guidance to the prescriber about which therapeutic alternative to select, the recommended equivalent dosage and regimen, as well as the recommended monitoring parameters. Data from the provincial monitoring report indicates that 28.6% of those patients potentially affected by the formulary change have switched to the fully covered RDP drug [4]. This is less than half of the proportion that was identified as switched in this study, supporting the alert as a useful aid in medication optimization.

While the majority of these individuals were on the modernized RDP drugs, a significant minority were not. For those who were not switched to the modernized RDP drugs, they could face unexpected increased medication costs at the pharmacy. Affordability of medications is already an issue for nearly 25% of all Canadian households [5], and approximately 10% of Canadians cannot afford to take their medications as prescribed [6]. While approximately two-thirds of Canadians have private drug insurance [7] and may not be affected by this change, private coverage is extremely variable, and as few as 2% of private drug plans parallel a provincial formulary [8]. This still presents an opportunity for cost reduction for equivalent outcomes.

Surprisingly, despite clear guidance from the Ministry of Health, professional medication expertise, and a reimbursement model in place, no pharmacist adaptations to adjust to the modernized RDP occurred either prior to the implementation of the changes or during the six months after the transition in the study population.

Continuing education programs were offered by the British Columbia Pharmacy Association and the University of British Columbia’s Continuing Pharmacy Professional Development team to support pharmacists with adapting prescriptions to address the formulary changes. However, enrolment for the education initiative did not begin until 30 November 2016 [9], the final day of the transition period, which may have affected the rate of adaptations. Professional culture, and personality traits of pharmacists practicing in the area may also have affected the rate of adaptations, however more studies are required in this area [10,11].

Non-dispensing clinical pharmacists working as integrated members of primary care clinics may be ideally placed to engage in clinical-wide quality improvement projects, such as adapting to changing formularies. Many EMRs have the ability to generate reports in order to quickly identify which patients are being prescribed certain medications, and interventions can be undertaken within the clinic to address these issues. Primary care clinics may be the preferred site for interventions, when compared to the pharmacy setting. However, in British Columbia, pharmacists working outside of a dispensary setting are unable to independently adapt prescriptions, as they do not have the original prescription, as they are presented to a licensed pharmacy. They must rely on prescriber collaboration to adjust prescription medications, despite having access to the full patient record (including lab results, specialist consults, and other pertinent medical information). Changes in legislation, allowing pharmacists working in this role to practice to their full scope, could have facilitated the substitution of drugs through this program. This would have resulted in greater efficiency and reduced drug expenditure.

Several limitations have been identified and must be acknowledged. This study addressed the six-month transition period, and the six-month period following the transition, and it is possible that adaptations occurred after the studied time period. This study also partially relied on pharmacist communication with the primary care clinics, and that this communication was captured in the clinic EMR to determine one of the results. It is possible in some instances that pharmacists were not following the required communication process, or that the clinics were not capturing the communication in the EMR, however this is believed to be very unlikely. Finally, it is not possible to precisely determine the extent to which the alert and guidance that is set within the EMR by the pharmacist facilitating the changes based on the data collected, although it is believed that this was a significant driver of the changes.

## 5. Conclusions

Adaptations of medications to reflect a change in provincial drug formulary supported by a clinical pharmacist, integrated within two clinics, occurred in 65% of the indicated cases; over double the provincial proportion of adaptations that occurred during the same timeframe. Direct collaboration between a clinical pharmacist working alongside NPs and FPs resulted in a higher proportion of the affected medications being changed when compared to standard practice, or community pharmacists alone.

## Figures and Tables

**Table 1:** Potential reference drug program (RDP) changes indicated and changes performed.

Drug Class	Number of Patients Prescribed a Drug in This Class	Number of Patients on the Modernized RDP Drug Prior to 1 December 2016 Transition	Potential RDP Adaptations Indicated (%)	RDP Adaptations by Family Pysicians or Nurse Practitioners during Transition Period	RDP Adaptations by FPs or NPs between 1 December 2016 and 31 May 2017	Total Number of Adaptations (%)
Angiotensin Converting Enzyme Inhibitors	229	203	26 (11%)	3	11	14 (53%)
Statins	233	213	17 (7%)	3	13	16 (94%)
Dihydropyridine Calcium Channel Blockers	83	70	12 (14%)	0	5	5 (41%)
Angiotensin Receptor Blockers	110	99	10 (9%)	0	7	7 (70%)
Total	655	585	65 (10%)	6	36	42 (65%)
